# Typhoid Fever due to Extended Spectrum *β*-Lactamase-Producing *Salmonella enterica* Serovar Typhi: A Case Report and Literature Review

**DOI:** 10.1155/2018/4610246

**Published:** 2018-02-15

**Authors:** Abdul Azeez Ahamed Riyaaz, Vindya Perera, Sabaratnam Sivakumaran, Nelun de Silva

**Affiliations:** ^1^Department of Internal Medicine, Dr. Neville Fernando Teaching Hospital, Malabe, Sri Lanka; ^2^Department of Microbiology, Faculty of Medicine, South Asian Institute of Technology and Medicine, Malabe, Sri Lanka

## Abstract

Emergence of cephalosporin-resistant strains of *Salmonella enterica* serovar Typhi is a cause of concern in the management of enteric fever. Cephalosporin resistance in *Salmonella* species is mainly due to the production of extended-spectrum *β*-lactamases (ESBLs). The majority of ESBLs in *Salmonella enterica* serovar Typhi are derivatives of the TEM, SHV, and CTX-M *β*-lactamase families. Of these, CTX-M appears to be predominant. This paper discusses the detection and molecular characterization of an ESBL-producing *Salmonella enterica* serovar Typhi strain isolated from a patient who was admitted to a private hospital in Sri Lanka. The three main types of *β*-lactamases such as TEM, SHV, and CTX-M were identified in this isolate. This case report from Sri Lanka contributes to the knowledge of the increasingly reported cases of typhoid fever due to *Salmonella enterica* serovar Typhi resistant to *β*-lactamase by ESBL production.

## 1. Introduction

The emergence of resistance to fluoroquinolones had led to the frequent use of azithromycin for empirical treatment of uncomplicated enteric fever and the use of third-generation cephalosporins as the first-line drug for intravenous treatments [1]. However, the recent emergence of cephalosporin-resistant strains of *Salmonella enterica* serovar Typhi is a cause for concern in the management of enteric fever. Cephalosporin resistance in *Salmonella* species is mainly due to the production of extended-spectrum *β*-lactamases (ESBLs). The majority of ESBLs in *Salmonella* are derivatives of the TEM, SHV, and CTX-M *β*-lactamase families.

The production of ESBL leads to multidrug resistance in *Salmonella enterica* isolates. Multidrug resistance (MDR) in *Salmonella enterica* has been described around the world and shown to be more common in *Salmonella* Typhi than in *Salmonella* Paratyphi [2, 3]. The geographic variations have been described for MDR *Salmonella* Typhi isolates in Asia. For example, some studies have shown higher prevalence of MDR phenotypes in India, Pakistan, and Vietnam when compared to those in China and Indonesia [4]. In developed countries such as the United States and Germany, *Salmonella* Typhi with MDR has been reported with travel to developing countries [5, 6].

In Sri Lanka, there are no published data on molecular characteristics of ESBL-producing *Salmonella enterica* serovar Typhi. This case reports the detection and molecular characterization of an ESBL-producing *Salmonella enterica* serovar Typhi strain isolated from a patient with typhoid fever who was admitted to a private hospital in Sri Lanka.

## 2. Case Presentation

An 18-year-old previously healthy male was admitted to our medical unit with a history of fever of 5-day duration. He had high-grade fever associated with generalized aches and frontal headache. He denied any respiratory or urinary symptoms. He mentioned that he had not passed faeces for the last three days but had developed nonbloody diarrhoea on the day of admission without any vomiting or abdominal pain. He denied any recent travel, but he had been eating from various food outlets for the past 2 weeks during his after-school cricket practice sessions.

On examination, he was not ill looking despite his temperature of 40°C. There were no pallor, icterus, lymphadenopathy, and skin rashes. Cardiovascular and respiratory examination was normal. Abdominal examination revealed a soft, nontender abdomen with a 3 cm nontender hepatomegaly and a 2 cm splenomegaly. His blood test showed a haemoglobin count of 14.8 g/dl, a white cell count of 2.8 × 10^9^/l (neutrophils 79% and lymphocytes 14%), and a platelet count of 97 × 10^9^/l. Dengue NS1 antigen and IgM against dengue virus detected by a commercial kit (Standard Diagnostics, Inc., Germany) were negative. C-reactive protein (CRP) was elevated more than 250 mg/dl in two subsequent samples. His liver function tests were normal except elevated liver transaminases (ALT 145 U/l and AST 118 U/l; ALT/AST normal range 8–40 U/L). An ultrasound scan of the abdomen confirmed mild hepatosplenomegaly. On days 2 and 3 of his admission, he continued to have high fever spikes, diarrhoea, and persistent thrombocytopenia with a high CRP.

Intravenous ceftriaxone 2 g daily was commenced. After 48 hours, the patients' clinical condition had not improved and the high fever spikes continued. After antibiotic susceptibility test results, his antibiotic was changed to imipenem 1 g 8 hourly. He showed clinical improvement after 48 hours, but the diarrhoea and fever settled only around the 10th day of treatment. Imipenem was continued for a total duration of 14 days. His platelet count, liver transaminases, and CRP returned to normal. A repeat blood and faeces culture performed on discharge was negative for *Salmonella enterica* serovar Typhi.

## 3. Laboratory Methods

The blood culture from the patient grew nonlactose fermenting colonies on MacConkey agar which were identified as *Salmonella enterica* serovar Typhi by standard laboratory methods and by the Rapid ID Test Kit (Oxoid).

The antibiotic-susceptibility results of the isolate performed according to CLSI disc diffusion method [2] showed resistance to antibiotics such as ampicillin, chloramphenicol, ciprofloxacin, cotrimoxazole, and co-amoxiclav. The isolate was susceptible to imipenem, meropenem, ertapenem, amikacin, and netilmicin but was resistant to cefotaxime, cefixime, cefepime, and aztreonam (Table 1). The resistance was further confirmed by determining the minimum inhibitory concentrations (MIC in *µ*g/ml) of the antibiotics to the isolate by VITEK® 2 system, and the results were as follows: ciprofloxacin >4, ceftriaxone >64, ceftazidime >64, imipenem <0.25, and ertapenem <0.5 (Table 2).

**Figure 1:**
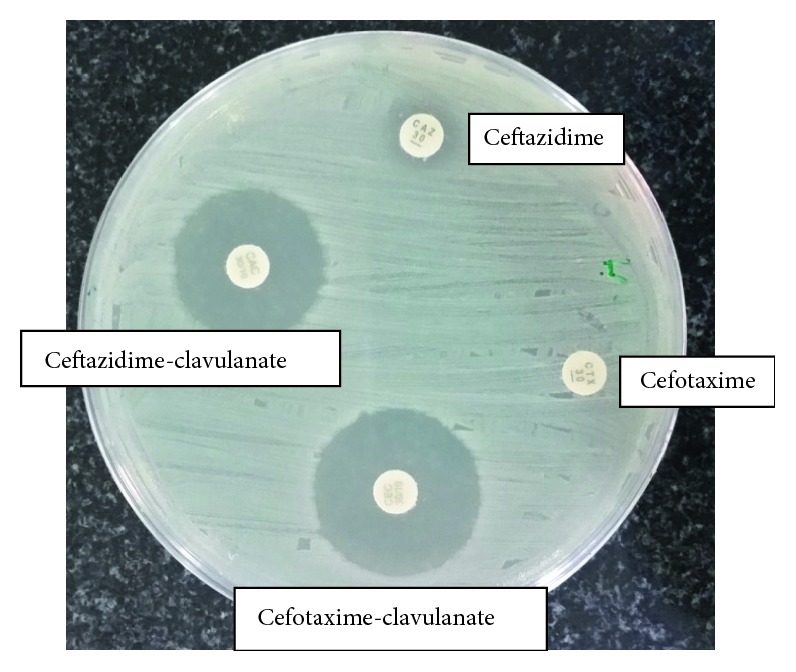
CLSI confirmatory test for ESBL production with >5 mm increase in zone diameters for combined discs of ceftazidime and cefotaxime with clavulanic acid versus the zone diameter of the agent when tested alone.

**Figure 2:**
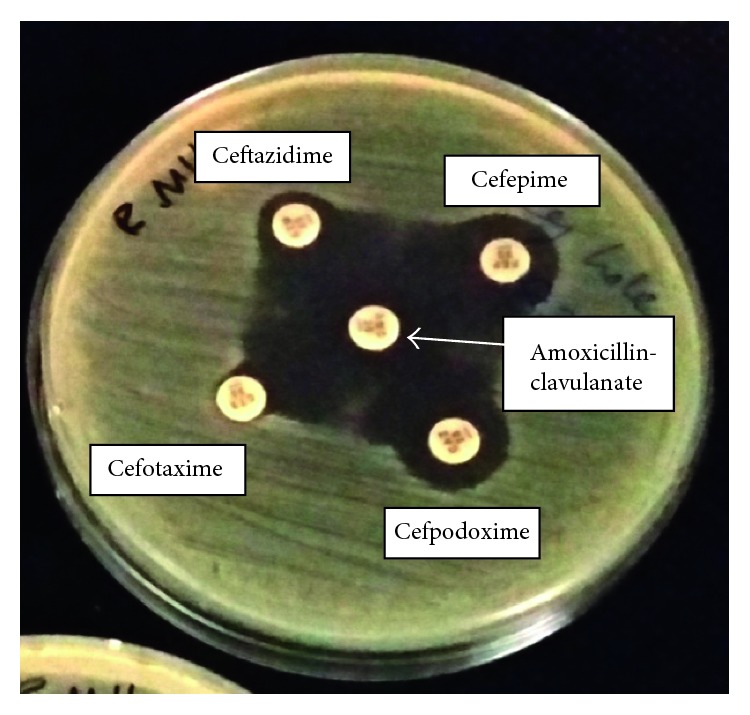
Modified double disc synergy test (MDDST) showing synergy between amoxicillin/clavulanate and four cephalosporins.

ESBL production of the isolate was screened according to CLSI breakpoints for cefpodoxime, ceftazidime, aztreonam, cefotaxime, and ceftriaxone. The combined disc test was done using ceftazidime and cefotaxime individually and in combination with clavulanic acid according to the Clinical and Laboratory Standards Institute (CLSI) guidelines to confirm ESBL production [7]. The isolate was further tested phenotypically with the modified double-disc synergy test (MDDST) by using a disc of amoxicillin-clavulanate along with four cephalosporins: cefotaxime, ceftriaxone, cefpodoxime, and cefepime [8]. Suggestive evidence of ESBL production was observed with reduced susceptibility for the five antimicrobial agents used in CLSI screening test. In the confirmatory test, >5 mm increase in zone diameters for the combined discs of ceftazidime and cefotaxime with clavulanic acid versus the zone diameter of the agent when tested alone was observed (Figure 1). In MDDST, a synergy between amoxicillin/clavulanate and four cephalosporins was observed which provided further evidence of ESBL production (Figure 2).

**Figure 3:**
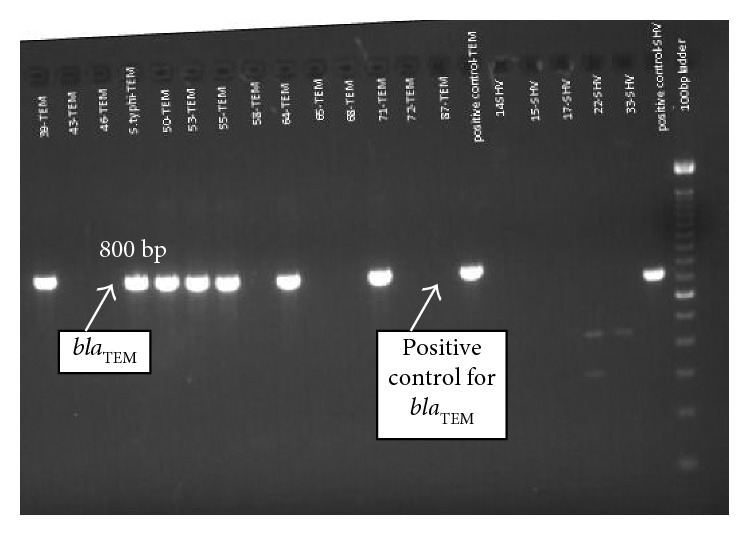
Gel image of PCR product showing *bla*_TEM_ gene.

**Figure 4:**
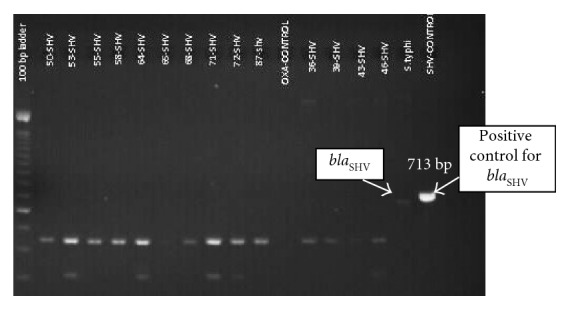
Gel image of the PCR product showing *bla*_SHV_ gene.

**Figure 5:**
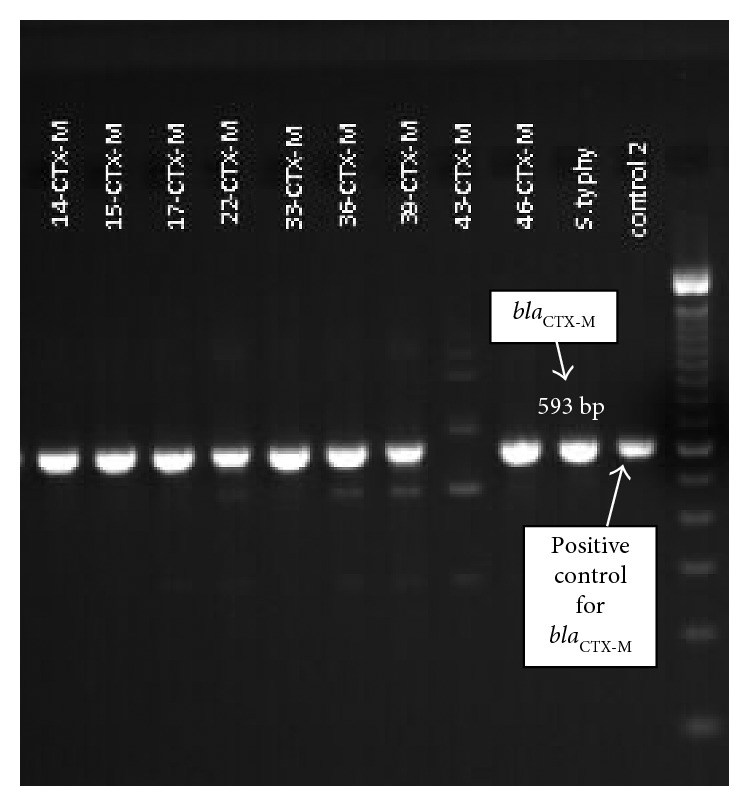
Gel image of the PCR product showing *bla*_CTX-M_ gene.

To detect ESBL genes, polymerase chain reaction (PCR) was performed as described previously [9, 10]. PCR results showed that the isolate harbored *bla*_TEM_ (Figure 3), *bla*_SHV_ (Figure 4), and *bla*_CTX-M_ (Figure 5) gene types.

## 4. Discussion

Enteric fever is one of the major health concerns in developing countries. *Salmonella* Typhi enters the lymphatic system after ingestion and then survives and replicates within macrophages, later on disseminating into the reticuloendothelial system. The common clinical features of enteric fever include fever, chills, abdominal pain, and appearance of rose spots. Leucopenia and anaemia are well-recognised haematological manifestations of enteric fever. Isolated thrombocytopenia is a rare finding in typhoid fever, and there are only few case reports found in the literature [11]. Our patient had both leucopenia and thrombocytopenia at presentation without anaemia, which is a common finding in febrile patients due to viral aetiology particularly dengue infection. Our patient presented during a dengue epidemic period where these findings could have been easily overlooked.

According to the literature, multidrug-resistant *Salmonella* Typhi had been documented more than *Salmonella* Paratyphi [12–14]. In our study, the isolate was resistant to all first-line antibiotics (i.e., ampicillin, chloramphenicol, cotrimoxazole, and ciprofloxacin).

The production of *β*-lactamases is a major factor that makes Gram-negative bacteria resistant to antibiotics [15]. TEM, SHV, and CTX-M are the main types of *β*-lactamases produced by these organisms that confer resistance to penicillins and cephalosporins. In this case, we identified the gene types responsible for the production of all the three main types of *β*-lactamases.

The TEM-type *β*-lactamase is commonly present in Enterobacteriaceae such as *Escherichia coli*, *Klebsiella pneumoniae*, and *Proteus mirabilis*, and SHV types were found to be common in *Klebsiella pneumonia* [16]. The CTX-M-type *β*-lactamases were found in *E. coli* and *Klebsiella* spp. Salmonella strains were documented to acquire these genes from *E. coli* and *Klebsiella* sp. [17]. Variants of these types of *β*-lactamases have conferred resistance only to penicillins and early cephalosporins as well as to second-, third-, and fourth-generation cephalosporins [18].

Several cases of *S. enterica* ser. Typhi resistant to *β*-lactamase by ESBL production have been reported in Asian countries. In the Philippines, a strain that harbors *bla*_SHV_ gene was identified in 2008 [19]. In Kuwait, the United Arab Emirates, and Bangladesh, there had been reports of ESBL-producing *Salmonella* Typhi with CTX-M genes [20, 21]. A study carried out in India in 2013 identified five multidrug-resistant isolates belonging to serotype *Salmonella* Typhi that were positive to TEM-1-type *β*-lactamase, but none of these isolates were positive for SHV- or CTX-M-type *β*-lactamase [22]. In the present case, we found a *S. enterica* serovar Typhi positive all TEM-, SHV-, and CTX-M-type *β*-lactamases.

The emergence of ESBL-producing *Salmonella* is a result of selective pressure imposed by the misuse of broad-spectrum antibiotics such as third-generation cephalosporins. Increased prevalence of these resistant salmonella will lead to failures of the current treatment practices. Continuous surveillance of the susceptibility profiles and improving infection control measures will help to control the spread of the infection with resistant strains. This case report describes a patient with typhoid fever caused by *Salmonella enterica* serovar Typhi strain that carried three major genes responsible for the production of ESBL that resulted in resistance to cephalosporins. To our knowledge, *Salmonella* Typhi resistant to *β*-lactamase by ESBL production and the genes encoding this resistance have not been described in Sri Lanka previously.

## Figures and Tables

**Table 1:** Antimicrobial susceptibility results of the isolate according to CLSI disc diffusion method.

Antimicrobial	CLSI disc diffusion method (interpretation)
Ampicillin	R
Chloramphenicol	R
Ciprofloxacin	R
Cotrimoxazole	R
Co-amoxiclav	R
Cefotaxime	R
Cefixime	R
Cefepime	R
Aztreonam	R
Imipenem	S
Meropenem	S
Ertapenem	S
Amikacin	S
Netilmicin	S

R, resistance; S, susceptible.

**Table 2:** The minimum inhibitory concentrations (MIC in *µ*g/ml) of the antibiotics to the isolate by VITEK 2 system.

Antimicrobial	MIC (mg/l)	Interpretation
Ciprofloxacin	>4	R
Ceftriaxone	>64	R
Ceftazidime	>64	R
Imipenem	<0.25	S
Ertapenem	<0.5	S

R, resistance; S, susceptible.
